# TFII-I/GTF2I regulates globin gene expression and stress response in erythroid cells

**DOI:** 10.1016/j.jbc.2025.108227

**Published:** 2025-01-24

**Authors:** Rukiye Nar, Matthew D. Gibbons, Leonardo Perez, John Strouboulis, Zhijian Qian, Jörg Bungert

**Affiliations:** 1 Department of Biochemistry and Molecular Biology, College of Medicine, Center for Epigenetics, Genetics Institute, UF Health Cancer Center, Powell-Gene Therapy Center, University of Florida, Gainesville, Florida, USA; 2 Department of Medicine, College of Medicine, Center for Epigenetics, Genetics Institute, UF Health Cancer Center, Powell-Gene Therapy Center, University of Florida, Gainesville, Florida, USA; 3 Red Cell Haematology, Comprehensive Cancer Centre, School of Cancer and Pharmaceutical Sciences, King’s College London, London, UK

**Keywords:** apoptosis, cell cycle, cell differentiation, gene regulation, stress response

## Abstract

Transcription factor TFII-I/GTF2I is ubiquitously expressed and has been shown to play a role in the differentiation of hematopoietic cells and in the response to various cellular stressors. We previously demonstrated that TFII-I acts as a repressor of adult **β**-globin gene transcription and positively regulates the expression of stress response proteins, including ATF3. Here we analyzed the function of TFII-I in TF-1 cells during erythroid differentiation and in response to cellular stress, including unfolded protein response, hypoxia, and oxidative stress. Ablation of TFII-I leads to mild changes in the cell cycle and proliferation of TF-1 cells. Importantly, TFII-I deficiency increased the expression of the adult **β**-globin gene with a concomitant reduction in the expression of the fetal γ-globin genes during erythropoietin-mediated erythroid differentiation of TF-1 cells. Furthermore, TFII-I regulates genes involved in stress response, including CHOP, Elongin A, ATF3, ATF4, and Grp78, and participates in the apoptotic response to stressors. In summary, the data provide further support for the role of TFII-I in stress response and the regulation of globin genes.

TFII-I is a multifunctional, ubiquitously expressed transcription factor that plays a role in the regulation of cell cycle and stress response genes (1, 2). The human *GTF2I* gene, which encodes TFII-I and several isoforms, is located on chromosome 7 (3, 4). Heterozygous deletion of a 1.5 to 1.8 Mb genomic region encompassing *GTF2I* and other genes causes Williams-Beuren syndrome (WBS), characterized by neurodevelopmental defects as well as craniofacial and cardiovascular abnormalities (5). Mutations in GTF2I are also common in thymomas, epithelial tumors of the thymus (6, 7).

TFII-I is an unusual transcription factor consisting of nuclear localization and basic region (BR) DNA-binding domains, a leucine zipper, and six so-called R-repeats, which resemble helix-loop-helix (HLH) domains (1, 2). Initially, TFII-I was found to interact with the initiator, a pyrimidine-rich sequence found at the transcription start sites (TSS) of many genes (8, 9). Subsequent studies demonstrated that TFII-I frequently interacts with E-box elements and other sequences that are associated with specific transcription factors (1, 2, 10). E-box elements are bound by HLH proteins, and TFII-I has been shown to interact with USF and MYC proteins (10, 11, 12, 13).

Deletion of the murine Gtf2i gene causes early embryonic lethality due in part to vascular defects (14). TFII-I activates or represses genes depending on the DNA-sequence context and interacting proteins. Consistent with these findings, TFII-I interacts with transcriptional co-repressors (HDAC and LSD1) or co-activators (chromatin remodeling factors, Elongin A, and topoisomerase II) (1, 13, 15). Interestingly, several data suggest that TFII-I interacts with CTCF and components of the cohesin complex (16, 17). CTCF has been shown to interact with topoisomerase II at the boundaries of topologically associated domains (TADs) (18). Furthermore, topoisomerase II associates with CTCF at fragile genomic sites prone to double-strand breaks (19). This is interesting in light of previous findings showing that TFII-I interacts with topoisomerase II and is implicated in DNA double strand repair (15, 20, 21).

How TFII-I contributes to cellular functions is not completely understood. TFII-I is known to regulate genes implicated in the response to cellular stress, including endoplasmic reticulum (ER) stress, as well as genes implicated in cell proliferation (1, 2). We previously found that TFII-I negatively regulates the expression of the adult β-globin gene (10, 22). Here, we analyzed the role of TFII-I in TF-1 erythroleukemia cells during differentiation and in response to cellular stress. TF-1 cells show many characteristics of erythroid cells and express almost no embryonic ϵ-globin at baseline (23, 24). TF-1 cells can be induced to differentiate into more mature erythroid cells using physiological inducers, including erythropoietin (EPO). The work presented here further supports a role for TFII-I in regulating the response to various cellular stressors and in regulating globin gene expression.

## Results

### Generation and selection of TFII-I depleted TF-1 cells

**Figure 1:**
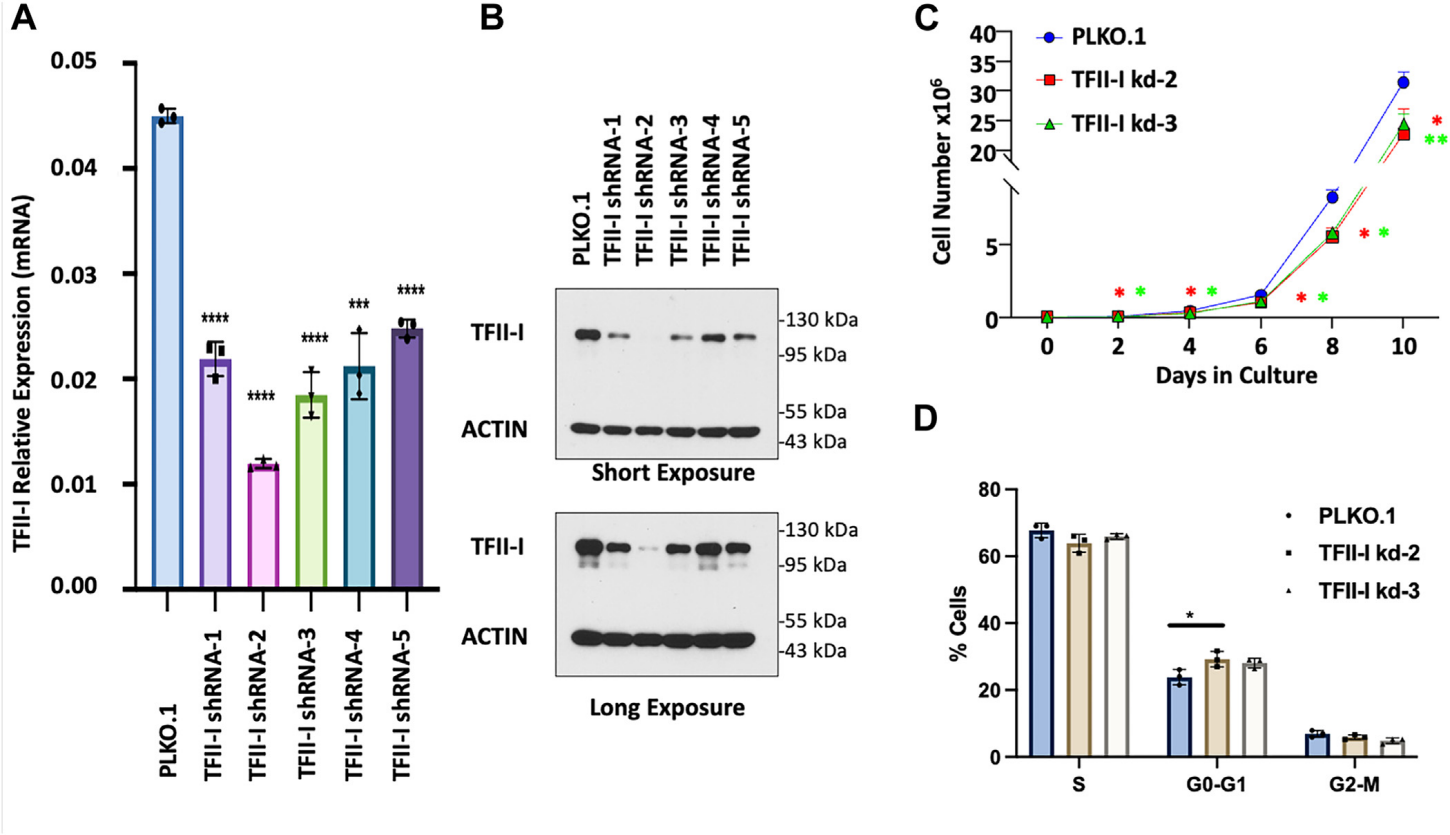
**Generation and cell cycle analysis of TFII-I deficient TF-1 cells.***A*, TF-1 cell clones harboring the control pLKO.1 vector or expressing shRNA targeted to the TFII-I mRNA (shRNA 1–5) were subjected to RT-qPCR with primers specific for TFII-I and GAPDH. TFII-I expression was normalized to GAPDH expression. *B*, Western blot analysis of TF-1 cell clones harboring the control pLKO.1 vector or expressing shRNA targeted to the TFII-I mRNA (shRNA 1–5). *C*, proliferation of TF-1 cells harboring the control pLKO.1 vector or expressing TFII-I shRNA-2 or -3. Life cells were counted every 48 h for 10 days. *D*, analysis of cell cycle distribution of TF-1 cells harboring the control pLKO.1 vector or expressing TFII-I shRNA-2 or -3. Cells were subjected to flow cytometry with APC-conjugated anti-BrdU and DAPI staining. Results are presented as the mean ± SD of three independent experiments (∗: *p* < 0.05, two-tailed Students *t* test).

Five shRNAs were selected to specifically target the human TFII-I/GTF2I mRNA. Control and TFII-I shRNA-expressing vectors (TFII-I shRNA 1-5) were stably transfected into TF-1 cells. After selection of stable clones, RNA was isolated and subjected to RT-qPCR analysis using primers specific for TFII-I and control GAPDH (Fig. 1*A*). The data show that TFII-I shRNAs-1, -2 and -3 were the most efficient in reducing TFII-I RNA abundance. This was confirmed by immunoblot analysis in which the expression of TFII-I was compared with that of β-actin (Fig. 1*B*). Expression of TFII-I mRNA and protein was strongly reduced in TF-1 cells expressing TFII-I shRNA-2. TF-1 cells stably expressing shRNA one and three also exhibited a reduction in TFII-I expression. Since RNA was slightly more reduced in cells expressing TFII-I shRNA-3, we continued the functional analysis with control TF-1 cells transfected with the empty vector (PLKOI.1) and TF-1 cells expressing TFII-I shRNA-2 and -3.

### TFII-I depletion affects the cell cycle and reduces TF-1 cell proliferation and colony formation

**Figure 2:**
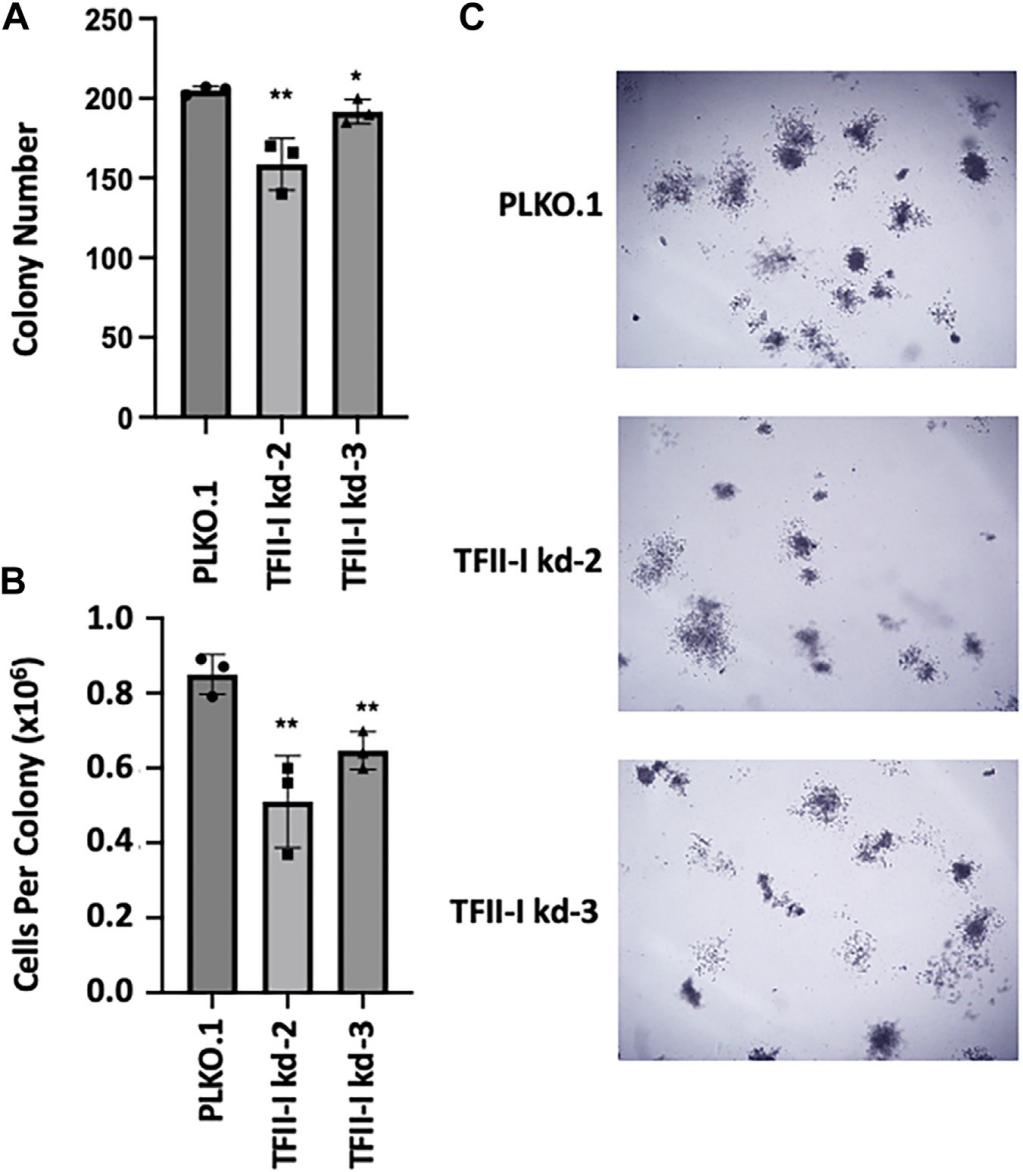
**TF-1 cell colony formation in TFII-I deficient cells.***A*, number of colonies per plate of TF-1 cells harboring the control pLKO.1 vector or vector expressing TFII-I shRNA-2 or -3. *B*, average cells per colony harboring the control pLKO.1 vector or vector expressing TFII-I shRNA-2 or -3. For *A* and *B*, results are represented as the mean + SD of three independent experiments (∗: *p* < 0.05; ∗∗: *p* < 0.009, two-tailed Students *t* test). *C*, representative images of colony formation of TF-1 cells harboring the control pLKO.1 vector or expressing TFII-I shRNA-2 or -3.

Depletion of TFII-I in both shRNA-2 and shRNA-3 expressing TF-1 cells resulted in a reduction of cell proliferation by about 30% (Fig. 1*C*). For cell cycle analysis, TF-1 control and shRNA-expressing cells were subjected to BrdU labeling and DAPI staining followed by flow cytometry analysis. The data show that TFII-I depletion increased the proportion of cells at the G0-G1 phase, which reached statistical significance in the TFII-I shRNA-2 expressing cells (Fig. 1*D*). These data suggest that the decrease in cell proliferation was caused by altered cell cycle progression, particularly a defect in the G1 to S-phase transition. In addition to reduced proliferation, depletion of TFII-I also caused a decrease in the number of TF-1 cell colonies (Fig. 2*A*) as well as in the number of cells within colonies (Fig. 2*B*). Representative images of control and shRNA expressing TF-1 cell colonies are shown in Figure 2*C*. In summary, TFII-I depletion reduces cell proliferation as well as the ability of TF-1 cells to form colonies.

### Gene expression changes in TFII-I depleted TF-1 cells

**Figure 3:**
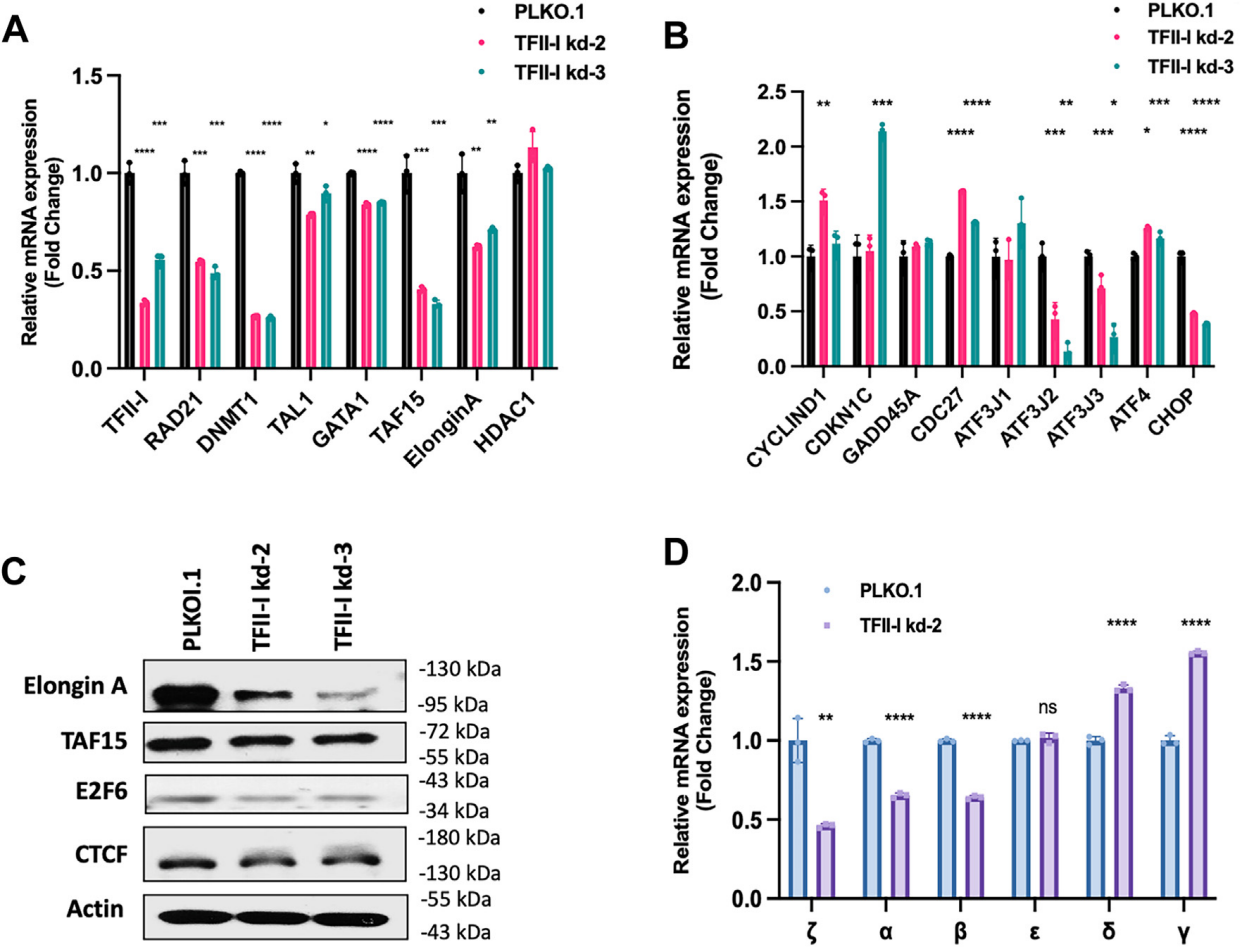
**Gene expression analysis in control and TFII-I deficient TF-1 cells.***A* and *B*, expression analysis of stress-response, erythroid-specific, and cell-cycle-specific gene in TF-1 cells harboring the control pLKO.1 vector or expressing TFII-I shRNA-2 or -3. Cells were subjected to RT-qPCR using primers specific for the genes listed on the x-axis; mRNA expression was normalized to that of GAPDH, and expression in empty vector (PLKO.1) harboring cells was set to 1 (∗∗∗: *p* < 0.0001; ∗∗∗: *p* < 0.001; ∗∗: *p* < 0.01; ∗: *p* < 0.05; two-tailed Students *t* test). *C*, Western blot analysis of Elongin A, TAF15, E2F6, CTCF, and β-actin in TF-1 cells harboring the pLKO.1 vector or expressing TFII-I shRNA-2 or-3. *D*, expression analysis of globin genes in TF-1 cells harboring the control pLKO.1 vector or expressing TFII-I shRNA-2. Cells were subjected to RT-qPCR using primers specific for the genes listed on the x-axis; mRNA expression was normalized to that of GAPDH, and expression in cells harboring the empty vector (PLKO.1) was set to 1. The error bars reflect the SEM from three independent experiments (∗∗∗∗: *p* < 0.001; ∗∗: *p* < 0.01; ns: nonsignificant, *p* > 0.05; two-tailed Students *t* test).

Next, we subjected TF-1 cells to gene expression analysis by RT-qPCR. We focused on a set of genes whose protein products are implicated in chromatin structure alterations and epigenetic modifications (RAD21, DNMT1, HDAC1), erythroid-specific gene regulation (GATA1 and TAL1), or shown to interact with TFII-I (TAF15 and Elongin A; Figure 3*A*) (15). In addition, we examined genes that encode proteins involved in cell cycle regulation (Cyclin D1, CDKN1C, GADD45A, CDC27), or in stress response (ATF3, ATF4, and CHOP; Fig. 3*B*). Overall, with a few exceptions, the results for the two different TFII-I shRNA expressing TF-1 cell clones (shRNA 2 and 3), were similar, meaning genes were either up or down-regulated in both clones, compared to cells harboring the vector control PLKO.1.

Expression of Rad21, DNMT1, TAF15, Elongin A, ATF3, and CHOP was consistently inhibited in the two TFII-I shRNA expressing clones (Fig. 3, *A* and *B*). We observed a small and consistent increase in the expression of ATF4 in TFII-I depleted cells (Fig. 3*B*). We previously examined the regulation of the stress-response gene ATF3 by TFII-I in K562 cells (15). Through streptavidin mediated pulldown of biotinylated TFII-I, we identified a TFII-I DNA-binding peak 5 Kb upstream of the ATF3 transcription start site. This binding peak was co-occupied by Pol II, and RT-qPCR experiments identified transcriptits originating from this TFII-I/Pol II occupied site, referred to as (ATF3 J1). In TF-1 cells, depletion of TFII-I did not alter non-coding transcription (ATF3 J1), but reduced expression of the ATF3 mRNA (ATF3 J2 and J3; referring to different exons of the ATF3 mRNA, Fig. 3*B*), consistent with our previous data from K562 cells. We previously found that TFII-I interacts with TAF15 and Elongin A (15). Interestingly, TFII-I depletion consistently reduced the expression of these genes in TF-1 cells (Fig. 3*A*). We also found reduced protein levels of TAF15 and Elongin A in the two TFII-I shRNA expressing clones (Fig. 3*C*). E2F6 protein levels were also reduced in the TFII-I deficient clones, consistent with previous findings (25). RAD21 is part of the cohesin complex and often associates with CTCF at boundary elements of topologically associated domains (TAD). Previous studies have shown that TFII-I interacts with CTCF and regulates its expression (17, 18). We did not see a change in CTCF protein expression in TF-1 cell clones expressing TFII-I shRNA (Fig. 3*C*). However, reduced levels of RAD21 mRNA suggest that TFII-I is implicated in the regulation of components of TAD boundaries.

Expression of the erythroid genes GATA1 and TAL1 was mildly reduced in the TFII-I depleted TF-1 clones (Fig. 3*A*) consistent with previous results (15). We did not detect a change in the expression of the histone deacetylase 1 (HDAC1) or the GADD45 genes in TFII-I shRNA expressing TF-1 clones (Fig. 3, *A* and *B*). Furthermore, we did not see consistent changes in the expression of the Cyclin D1 and CDKN1C genes in the TFII-I depleted TF-1 cell clones (Fig. 3*A*). Overall, the data are consistent with our previous observations in TFII-I depleted K562 cells, and reveal novel aspects of TFII-I function, including its implication in the regulation of CHOP, TAF15, Elongin A, and RAD21 genes.

Expression of CDC27 was consistently upregulated in the two TFII-I shRNA-expressing TF-1 cell clones (Fig. 3*B*). The *CDC27* gene is likely a direct target of TFII-I as we have previously identified several TFII-I binding peaks in the *CDC27* gene locus (25). CDC27 is part of the anaphase-promoting complex that mediates ubiquitin-dependent proteolysis of b-type cyclins (26). Cyclin B normally accumulates during the cell cycle until its degradation before mitosis (27). Thus increased CDC27 levels could contribute to the cell-cycle changes observed in TFII-I depleted TF-1 clones (Fig. 1*D*).

We also analyzed the expression of all human globin genes in undifferentiated control and TFII-I shRNA-2 expressing TF-1 cells. We observed mild reductions in the expression of the embryonic ζ-globin gene as well as the adult α- and β-globin genes, and a mild increase in the expression of the fetal γ-globin and adult δ-globin genes (Fig. 3*D*). The mild increase in γ-globin expression in the undifferentiated cells in TFII-I depleted cells could be related to the mild increase in ATF4 expression (Fig. 3*B*). ATF4 has been shown to increase γ-globin expression by stimulating the expression of transcription factor MYB (28).

### TFII-I regulates globin gene expression during erythroid differentiation of TF-1 cells

**Figure 4:**
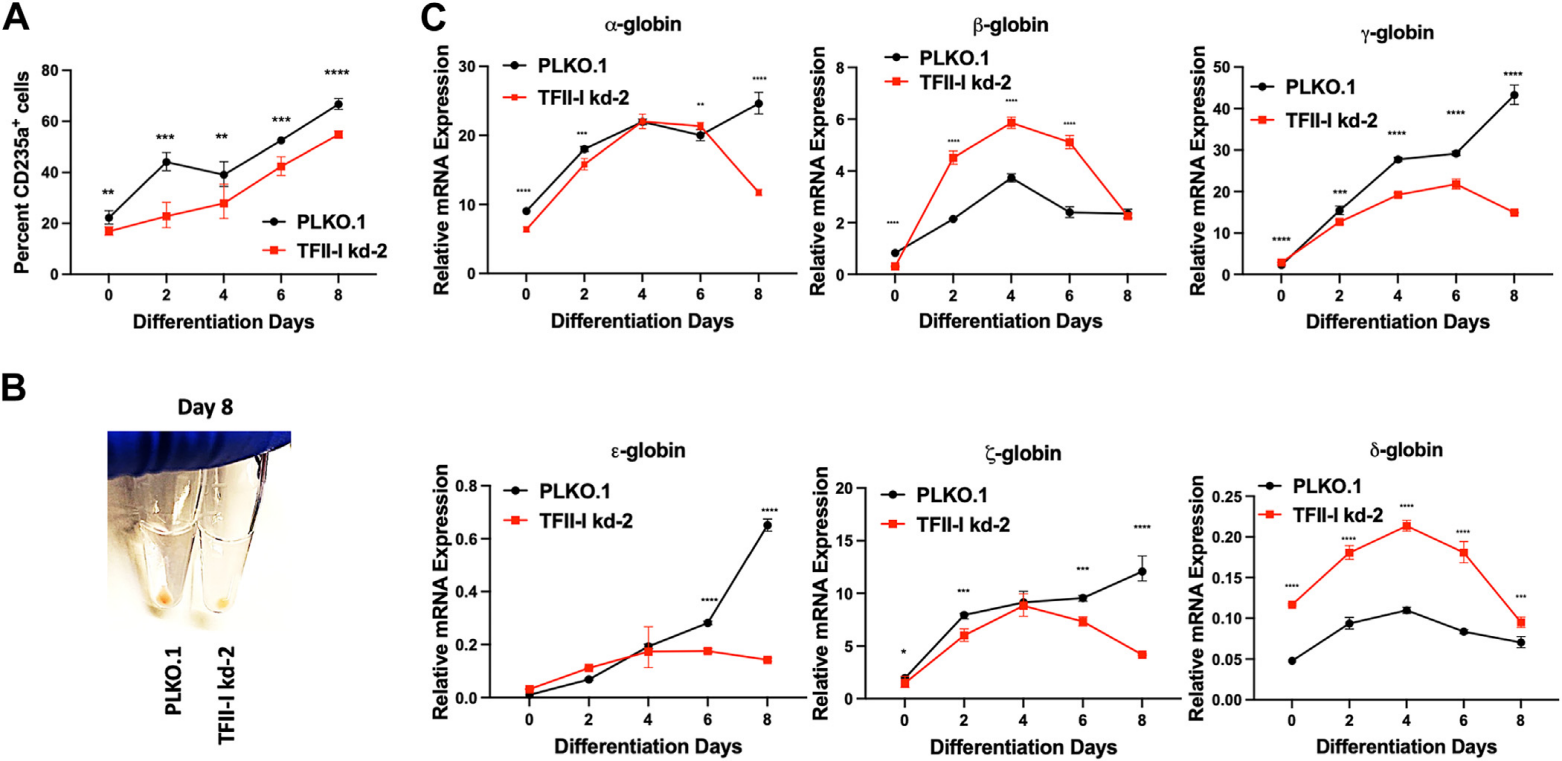
**Erythroid differentiation and globin gene expression in control and TFII-I deficient differentiating TF-1 cells.***A*, percentages of CD235a^+^ cells in control and TFII-I knockdown cells at various time points of culture with EPO. The experiment has been repeated three times (n = 3) and the error bars reflect the standard error of the mean (∗∗∗∗*p* < 0.0001; ∗∗∗*p* < 0.001; ∗∗*p* < 0.05; two-tailed Students *t* test). *B*, hemoglobinization of TF-1 cells harboring the control pLKO.1 vector or expressing TFII-I shRNA-2. Cells were pelleted and photographed after 8 days in culture with erythropoietin. *C*, relative globin gene expression during erythropoietin-induced erythroid differentiation of TF-1 cells harboring the control pLKO.1 vector or expressing TFII-I shRNA-2. Cells were subjected to RT-qPCR using primers specific for the genes listed on the x-axis; mRNA expression was normalized to that of GAPDH. The error bars reflect the SEM from three independent experiments (∗: *p* < 0.05, ∗∗: *p* < 0.001, ∗∗∗: *p* < 0.0003, ∗∗∗∗: *p* < 0.0001; two-tailed Students *t* test).

TFII-I has previously been implicated in the regulation of globin genes, and conditional ablation of TFII-I expression in hematopoietic cells in mice led to an increase in expression of the adult β-globin gene, suggesting that TFII-I is a repressor of adult β-globin expression (10, 22, 29). We induced differentiation of TF-1 cells along the erythroid lineage and examined the expression of all human globin genes in control and TFII-I shRNA-2 expressing cells. Erythroid differentiation was monitored by determining the percentage of CD235a (glycophorin A) positive cells by flow cytometry over a period of 8 days. TFII-I deficiency caused a reduction in the number of glycophorin A positive cells throughout the 8-day time period (Fig. 4*A*) and also reduced hemoglobinization of differentiated TF-1 cells (Fig. 4*B*; cells are significantly less red at day 8).

The TF-1 cells expressed relatively high levels of α-, γ-, and ζ-globin genes throughout the course of erythroid differentiation (Fig. 4*C*). Expression of ϵ- and δ-globin was low in these cells, while β-globin was expressed at intermediate levels. Depletion of TFII-I caused an increase in expression of adult β- and δ-globin genes from Day 2 to Day 6 of differentiation compared to the control cells. The increase in expression of the adult globin genes was accompanied by a decrease in fetal γ-globin expression at Days 4 and 6 of differentiation. At Day 8 of differentiation, expression of all globin genes decreased in TFII-I depleted cells, while expression of α-, γ-, ϵ- and ζ-globin genes increased in the control cells. The decrease in expression of the fetal γ-globin gene could be a consequence of increased adult β-globin expression. The data continue to demonstrate that TFII-I is a repressor of adult β-globin gene expression in differentiating erythroid cells.

### Depletion of TFII-I alters the response to cellular stress in TF-1 cells

**Figure 5:**
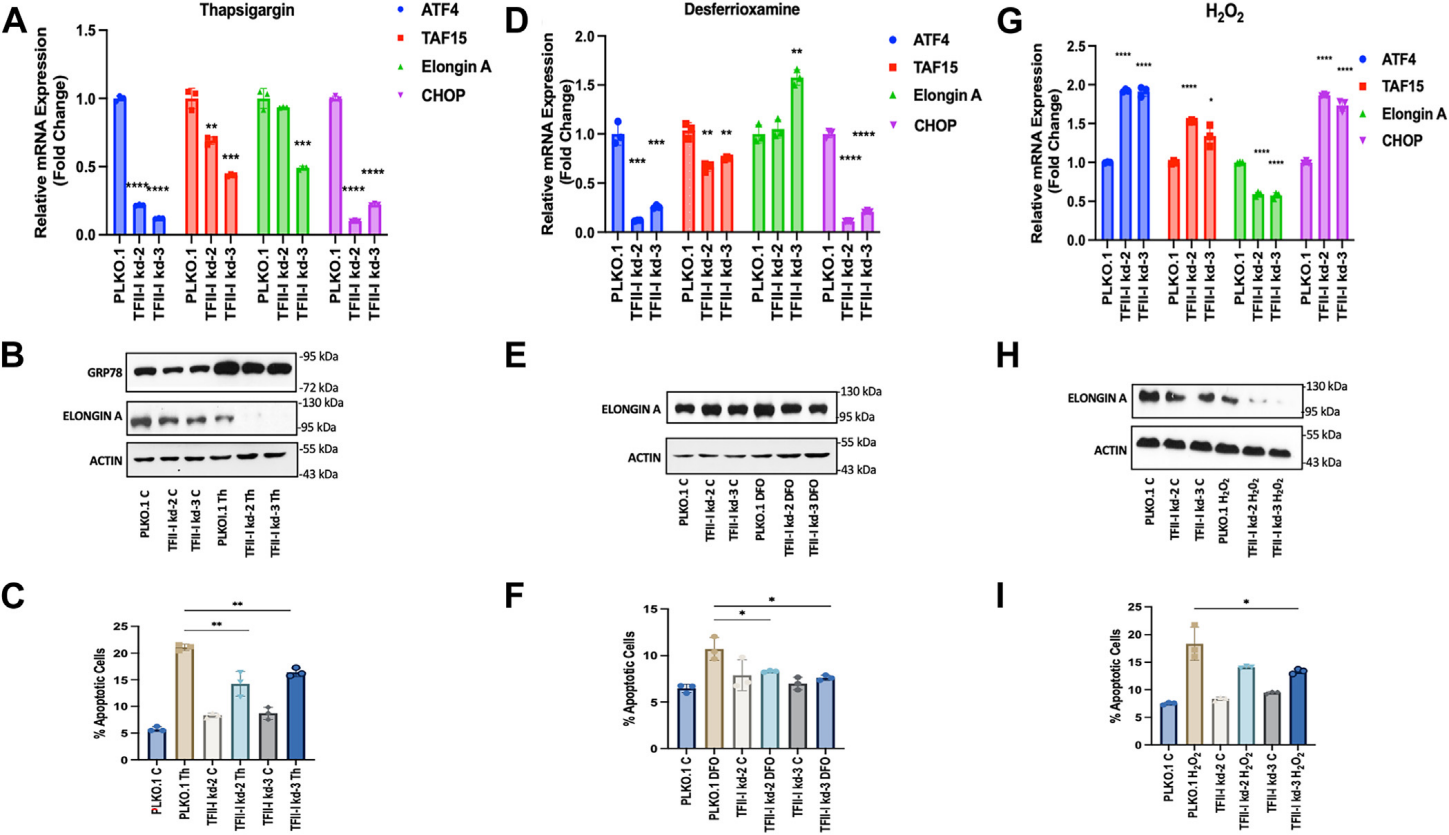
**Gene expression and apoptosis analysis in control and TFII-I deficient TF-1 cells during response to hypoxia as well as ER and oxidative stress.***A*–*C*, cells were treated for 8 h with 100 nM thapsigargin. Control groups were treated with DMSO. After 8 h, cells were collected for analysis of mRNA expression, western blotting, and apoptosis. *A*, gene expression analysis of TF-1 cells harboring the control pLKO.1 vector or expressing TFII-I shRNA-2 or -3. Cells were subjected to RT-qPCR using primers specific for the genes listed on the x-axis; mRNA expression was normalized to that of GAPDH. The experiments have been repeated three times (n = 3) and the error bars reflect the standard error of the mean (∗∗∗∗: *p* < 0.0001; ∗∗∗: *p* < 0.001; ∗∗: *p* < 0.01; two-tailed Students *t* test). *B*, Western blot analysis for GRP78, Elongin A and Actin in TF-1 cells harboring the pLKO.1 vector or expressing TFII-I shRNA-2 or -3 in control (C) and thapsigargin treated cells (Th). *C*, analysis of apoptotic frequency of TF-1 cells harboring the control pLKO.1 vector or expressing TFII-I shRNA-2 or -3 (∗∗: *p* < 0.01; two-tailed Students *t* test). *D*–*F*, cells were treated with DFO at a final concentration of 100 μM, while control groups were treated with saline. After 24 h, cells were collected for analysis of mRNA expression, western blotting, and apoptosis. *D*, gene expression analysis of TF-1 cells harboring the control pLKO.1 vector or expressing TFII-I shRNA-2 or -3. Cells were subjected to RT-qPCR using primers specific for the genes listed on the x-axis; mRNA expression was normalized to that of GAPDH. The experiments have been repeated three times (n = 3) and the error bars reflect the standard error of the mean (∗∗∗∗: *p* < 0.0001; ∗∗∗: *p* < 0.001; ∗∗: *p* < 0.01; two-tailed Students *t* test). *E*, Western blot analysis for Elongin A and β-Actin in TF-1 cells harboring the pLKO.1 vector or expressing TFII-I shRNA-2 or -3 in DFO treated cells (DFO). *F*, analysis of apoptotic frequency of TF-1 cells harboring the control pLKO.1 vector or expressing TFII-I shRNA-2 or -3 in control (C) and DFO-treated cells (∗: *p* < 0.05; two-tailed Students *t* test). *G*–*I*, cells were incubated in the absence (C) and presence of 150 μM H_2_O_2_ for 24 h. After 24 h, cells were collected for analysis of mRNA expression, western blotting, and apoptosis. *G*, Gene expression analysis of TF-1 cells harboring the control pLKO.1 vector or expressing TFII-I shRNA-2 or -3. Cells were subjected to RT-qPCR using primers specific for the genes listed on the x-axis; mRNA expression was normalized to that of GAPDH. The experiments have been repeated three times (n = 3) and the error bars reflect the standard error of the mean (∗∗∗∗: *p* < 0.0001; ∗:*p* < 0.05; two-tailed Students *t* test). *H*, Western blot analysis for Elongin A and β-Actin in TF-1 cells harboring the pLKO.1 vector or expressing TFII-I shRNA-2 or -3 in control (C) and H_2_O_2_ treated cells (H_2_O_2_). *I*, analysis of apoptotic frequency of TF-1 cells harboring the control pLKO.1 vector or expressing TFII-I shRNA-2 or -3 in control (C) and H_2_O_2_ treated cells (∗: *p* < 0.05; two-tailed Students *t* test).

We subjected TF-1 control cells and TF-1 cells expressing TFII-I shRNA-2 or -3 to various cellular stressors and examined the expression of stress-response genes and apoptosis. Thapsigargin induces the unfolded protein response leading to what is referred to as endoplasmic reticulum (ER) stress (30). Several proteins are induced during ER stress, including the glucose-response-protein 78 kDa (GRP78), an ER associate chaperone (31). TFII-I deficiency consistently reduced the expression of TAF15 and C/EBP-homologous protein (CHOP) in TF-1 cells (Fig. 5*A*). CHOP is a transcription factor that plays an important role in the response to ER stress and in stress-induced apoptotic pathways (32, 33). The reduction of Elongin A mRNA in TF-1 cells expressing shRNA was only statistically significant in the TFII-I shRNA-3 expressing clone (Fig. 5*A*). However, Elongin A protein levels were reduced in both TFII-I shRNA-expressing clones (Fig. 5*B*). Expression of GRP78 was induced upon treatment of TF-1 cells with thapsigargin, and this was reduced in the two TFII-I deficient TF-1 cell lines (Fig. 5*B*). The apoptotic frequency was increased in TF-1 cells exposed to thapsigargin; however, this increase was blunted in cells expressing TFII-I sRNA −2 and −3 (Fig. 5*C*), indicating that TFII-I exhibits pro-apoptotic activity during ER stress.

Hypoxia also causes cellular stress, which is particularly relevant to erythroid cells (34, 35). Desferrioxamine (DFO) is an iron chelator that increases the expression of HIF-1α, a hallmark of hypoxia (36). In contrast to thapsigargin-treated cells, TFII-I depletion did not cause a strong reduction of neither TAF15 nor Elongin A mRNA in TF-1 cells incubated with DFO (Fig. 5*D*). However, Elongin A protein levels were decreased in DFO-treated cells expressing TFII-I shRNA (Fig. 5*E*). Consistent with thapsigargin-treated cells there was a strong reduction in ATF4 and CHOP expression in DFO-treated cells. Also consistent with thapsigargin-treated cells, DFO increased apoptosis in TF-1 control cells (Fig. 5*F*), although the increase in apoptosis was not as pronounced as the increase in response to thapsigargin. The increase in apoptosis was not seen in DFO-treated cells expressing TFII-I shRNA-2 and -3, showing that TFII-I has pro-apoptotic activity under these conditions as well.

Oxidative stress was induced by treatment of cells with H_2_0_2_ (37). Under conditions of oxidative stress, there was an increase in the expression of CHOP, ATF4, and TAF15, while the expression of Elongin A declined in the two TFII-I deficient TF-1 cell clones (Fig. 5*G*). Reduced Elongin A expression was confirmed by immunoblotting experiments (Fig. 5*H*). Similar to DFO-treated cells, oxidative stress increased the frequency of apoptosis, and this increase was blunted in TFII-I shRNA-expressing cells, although this was only statistically significant in cells expressing TFII-I shRNA-3 (Fig. 5*I*).

**Figure 6:**
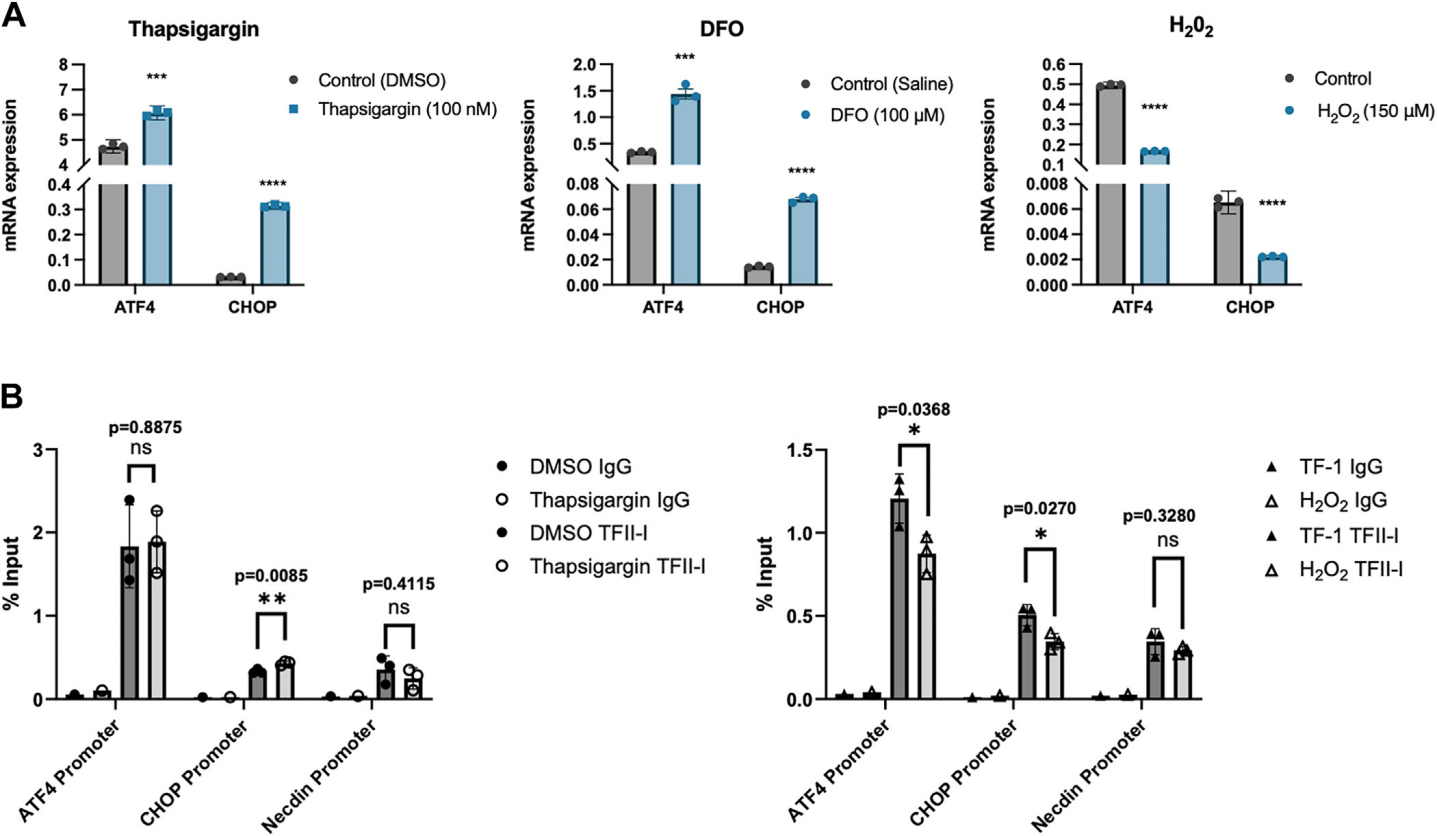
**Expression of the ATF4 and CHOP genes and interactions with TFII-I in response to hypoxia and/or ER and oxidative stress.** TIF-1 cells were subjected to cellular stress with thapsigargin, DFO, or H_2_O_2_, as indicated. *A*, expression analysis of ATF4 and CHOP in control and stress-induced cells. The mRNA was isolated, converted to cDNA, and subjected to qPCR using primers specific for the ATF4 or CHOP genes, as indicated. The data were normalized to GAPDH and the error bars reflect the standard error of the mean of three independent experiments (∗∗∗∗: *p* < 0.0001; ∗∗∗: *p* < 0.001; two-tailed Students *t* test). *B*, interactions of TFII-I with the ATF4, CHOP, and necdin promoter, in control and stress-induced cells. Control or stress-induced TF-1 cells were crosslinked and subjected to ChIP and qPCR using primers specific for the ATF4, CHOP, or necdin promoter, as indicated. The data are shown as % input and the error bars reflect the mean of three independent experiments (∗∗: *p* < 0.01; ∗: *p* < 0.05; two-tailed Students *t* test).

The differential changes in ATF4 and CHOP expression after TFII-I depletion in response to thapsigargin and DFO *versus* H_2_0_2_ are interesting. We examined changes in ATF4 and CHOP expression during the stress response (Fig. 6*A*). The data show that treatment of TF-1 cells with thapsigargin or DFO led to a relatively small increase in ATF4 expression and a pronounced increase in CHOP expression. In contrast, treatment with H_2_O_2_ decreased both ATF4 and CHOP expression. It should be noted that baseline expression of the ATF4 and CHOP genes was different in the controls including DMSO, saline, or no addition, respectively. There are no ChIP-seq data available for TFII-I in TF-1 cells. The TFII-I ChIP-seq data in K562 cells reveal a noisy background and there is no clear binding of TFII-I to either the ATF4 or CHOP promoter region. We carried out ChIP-qPCR experiments to examine the binding of TFII-I to the ATF4 and CHOP promoters in untreated control and thapsigargin or H_2_O_2_ treated cells. The data show that TFII-I interacted with the ATF4 promoter (Fig. 6*B*). Binding at the CHOP promoter was not significantly increased over binding to the neuronal necdin promoter. There was no change in binding to the ATF4 promoter in thapsigargin treated cells but a small and statistically significant increase in binding at the CHOP promoter. In contrast, H_2_O_2_ treatment caused a decrease in the binding of TFII-I to both ATF4 and CHOP promoters, without significant changes in the binding to the necdin promoter. These data suggest that expression of ATF4 and CHOP is regulated differently in thapsigargin *versus* H2O2-treated TF-1 cells.

## Discussion

Transcription factor TFII-I is a multifunctional protein that is structurally more complex than most transcription factors (1, 2). Its nuclear/cytoplasmic location is regulated by phosphorylation and TFII-I appears to function in both compartments. In the nucleus, TFII-I associates with chromatin and regulates transcription and DNA repair (20). The global knock-out of TFII-I results in early embryonic lethality due in part to defects in the vasculature (14, 38). Hemizygous germ line mutations of TFII-I are associated with neuronal and craniofacial abnormalities (3, 5). Mutations of TFII-I have also been associated with thymic epithelial tumors, including thymomas (6, 7).

How TFII-I regulates transcription is not completely understood. TFII-I was originally shown to bind the pyrimidine-rich initiator and to facilitate the formation of RNA polymerase II (Pol II) transcription complexes on TATA-deficient promoters (8, 9). The binding of TFII-I to the initiator has been verified by subsequent studies (10). These studies led to the realization that TFII-I can function as an activator or repressor of transcription. Accordingly, TFII-I has been shown to interact with positive and negative co-regulatory proteins (1, 2, 15). We previously demonstrated that TFII-I is a negative regulator of adult β-globin expression (10, 22, 29). Here we show that TFII-I deficiency increases adult β- and δ-globin expression and at the same time decreases expression of the fetal γ-globin genes. The decrease in γ-globin expression is likely a consequence of increased β-globin expression as both genes are regulated competitively by the locus control region (LCR) (39, 40). ATF4 has been implicated in both positive and negative regulation of fetal hemoglobin and we show that TFII-I deficiency slightly increases ATF4 and γ-globin expression in undifferentiated TF-1 cells (28, 41). The repression of adult β-globin expression may be mediated by binding to the initiator element and the prevention of TFIID recruitment to the basal promoter (10, 22). Alternatively, a recent study found that TFII-I interacts with the initiator and recruits negative elongation factor-A (NELF-A), which leads to defects in the pause release of Pol II (42). Previously, we showed that TFII-I interacts directly or indirectly with Elongin A, a protein complex that suppresses the transcription pausing of Pol II (15). This suggests that TFII-I is capable of regulating both Pol II pausing and elongation through differential interactions with positive and negative elongation factors. How this is regulated in a gene-, cell-, or environmental-specific manner remains to be investigated.

We and others previously demonstrated that TFII-I regulates genes involved in the unfolded protein response, including ATF3 (15, 31). In the current study, we show that TFII-I positively regulates ATF4, TAF15, CHOP, and GRP78 under conditions of ER stress. Thus, TFII-I regulates the major players involved in the response to ER stress. TFII-I may regulate these genes directly or indirectly. In support of a direct mode of regulation found that TFII-I interacts with the promoter of the ATF4 gene. ATF4 is known to initiate the response to ER stress by increasing the expression of CHOP and GRP78 (43).

Gene expression patterns were similar in DFO-treated cells compared to thapsigargin-treated cells, showing that TFII-I deficiency caused a decrease in the expression of ATF4, CHOP, and TAF15, although the effect on TAF15 was mild. The reduced expression of ATF4 and CHOP is consistent with previous results showing that ATF4 mediates the response to ER stress as well as hypoxia (44). Similar to thapsigargin-treated cells, the negative effect of TFII-I deficiency on Elongin A in DFO-treated cells is restricted mostly to protein levels. The situation was different under conditions of oxidative stress. In cells treated with H_2_O_2_, TFII-I deficiency increased the expression of ATF4, CHOP, and TAF15, but decreased the expression of Elongin A. Despite the differential expression of ATF4 and CHOP in TFII-I depleted cells in response to the different stressors, under all stress conditions examined here, TFII-I deficiency blunts the apoptotic response. This suggests that TFII-I exhibits pro-apoptotic activity in stressed cells. In contrast to these findings, TFII-I was shown to exhibit anti-apoptotic activity during the development of neurons (45). For ER and hypoxia-stressed cells, the pro-apoptotic effect of TFII-I may be mediated by CHOP, which is reduced in TFII-I-depleted cells. CHOP has been shown to be involved in apoptotic pathways (33). However, the pro-apoptotic effect of TFII-I in H_2_O_2_ treated cells is likely regulated by other activities.

TFII-I exerts functions in both the nucleus and cytoplasm. For example, TFII-I was shown to negatively regulate agonist-induced calcium entry (46). This is important to note, as thapsigargin raises cytosolic calcium concentration and depletes ER calcium stores, thus inducing the unfolded protein response (30). Hypoxia also regulates calcium transients and signaling (47). This suggests that TFII-I may contribute to the stress response through nuclear and cytoplasmic activities. Interestingly, it was shown that the protein deglycase DJ-1, implicated in Parkinson’s disease, interacts with TFII-I in the cytosol and prevents its nuclear translocation, which attenuates the stress response (48).

Taken together, the analysis of different cellular stressors revealed that the effect of TFII-I on gene expression varies while the effect on apoptosis was similar under different conditions. Changes in CHOP gene expression correlated with ATF4 expression under all conditions, suggesting that TFII-I deficiency-mediated regulation of CHOP expression is governed by ATF4 levels.

## Experimental procedures

### TF-1 cell culture, erythroid differentiation, and stress induction

The TF-1 erythroleukemia cell line was obtained from the American Type Culture Collection (ATCC). TF-1 cells were incubated in RPMI 1640 medium (Gibco) containing 10% fetal bovine serum, 1% penicillin-streptomycin solution (Sigma), and 2 ng/ml of granulocyte-macrophage colony-stimulating factor (GM-CSF; PeproTech) for long term growth at 37 °C with 5% CO2. Cells were passaged every 2 to 3 days. To induce erythroid differentiation, TF-1 cells were grown for the indicated time in RPMI 1640 medium supplemented with 10% FBS, 50U/ml-50μg/ml Penicillin-Streptomycin, and 2ng/ml human recombinant GM-CSF. The cells were pelleted, washed two times in non-supplemented RPMI to withdraw GM-CSF, and then cultured overnight in media lacking GM-CSF. After GM-CSF starvation, cells were pelleted and washed twice with RPMI. 2 × 10^5^ cells/ml were resuspended and differentiated in RPMI-1640 medium with 2 IU/ml recombinant human erythropoietin (Human EPO, Acro) and without GM-CSF for a total of 8 days. Cells were spun down, and fresh medium was added every 2 days of differentiation. Erythroid differentiation was assessed by flow cytometry. Thapsigargin (Tg) was purchased from MedChemExpress (HY-13433 MCE). TF-1 cells (5 × 10^5^ cells/well) were plated in 24-well plates. Cells were treated for 8 h with 100 nM Tg. Control groups were treated with DMSO. After 8 h, cells were collected for apoptosis, western blotting, and RNA isolation. Desferrioxamine (DFO) mesylate was purchased from EMD Millipore (252750). Cells were plated at 5 × 10^5^ cells per well in six-well plates in a starvation medium of 0.2% (v/v) FBS in RPMI 1640. On day 2, cells were treated with DFO at a final concentration of 100 μM DFO. Control groups were treated with saline. After 24 h, cells were collected for apoptosis, western blotting, and RNA isolation. Hydrogen peroxide (H_2_0_2_) was purchased from Thermo Fisher (202460010). TF-1 cells (5 × 10^5^ cells/well) were plated in 24-well plates. After stimulation with 150 μM H_2_O_2_ cells for 24 h, cells were collected for proliferation assay, apoptosis, western blotting, and RNA isolation.

### Lentiviral constructs, packaging, and viral transduction

TRC Lentiviral pLKO.1 plasmid expressing empty vector control shRNA and TFII-I shRNAs targeting different specific sequences of the TFII-I coding region were purchased from Dharmacon (TRCN0000019314, TRCN0000019315, TRCN0000019316, TRCN0000019317, and TRCN0000019318). Third-generation self-inactivating lentiviral transfer vectors, the plasmids encoding the different envelope proteins (pMD.G), and the packaging vector pCMV-ΔR8.91 were co-transfected overnight into 60 to 70% confluent 293T cells (human Ad5-E1 transformed embryonic kidney cells expressing the Simian Virus 40 (SV40) T-antigen gene, obtained from ATCC) using the polyethylenimine (PEI) method. Briefly, plasmids were mixed at the following concentrations: 6.5 μg pCMV-ΔR8.91, 3.5 μg pMD.G and 10 μg of lentiviral control or shRNA expressing TFII-I plasmids, followed by the addition of 80 μl polyethylenimine to Opti-MEM I (Gibco; Thermofisher Scientific catalog #31985070) at a total volume of 1 ml. The content of the tube was gently mixed, incubated at room temperature (RT) for 18 min, and added to the culture medium (Dulbecco’s Modified Eagle Medium, Gibco|Thermofisher Scientific, catalog #41966052, supplemented with 10% FBS and 1% penicillin-streptomycin solution (Sigma)) in a 10 cm dish containing 293T cells. After overnight incubation (37 °C/5% CO_2_) the culture medium was replaced and the supernatant was collected after 48 and 72 h post-transfection, centrifuged for 5 min at 845*g* at RT, and subsequently passed through 0.45 μm pore-sized filters, aliquoted and stored at −80 °C. For viral transduction, TF-1 cells (0.75 × 10^6^ per well) were plated in a 24-well culture dishes and spin infected with viral supernatants containing PLKO.1 (empty vector) or TFII-I shRNA expressing virus (2 ml per well) supplemented with 8 μg/ml polybrene at 2000 rpm and 32 °C for 180 min by using an Eppendorf 5810/5810 R centrifuge. The medium was removed and replaced with fresh medium overnight, followed by a second spin-infection. After 24 h, puromycin was added for the selection and cells were cultured for 3 days.

### Cell proliferation and colony-forming unit (CFU) assays

Control or TFII-I KD cells were seeded at a density of 2 × 10^4^ cells per well in a 96-well plate. Cells (10 μl) were removed, and stained with trypan blue, and unstained cells were counted as viable. Every 2 days the medium was changed, and cells were diluted with fresh medium; the cell number was multiplied by the dilution factor. All experiments were performed in triplicate. TF-1 cells were counted for cell viability and an equal number of live cells (1 × 10^3^ cells/ml) were plated in methylcellulose medium containing 1% methylcellulose (M3134; StemCell Technologies, Inc), 30% fetal bovine serum, 2% penicillin and streptomycin, 3.0 ng/ml granulocyte colony-stimulating factor in the presence of puromycin in 24-well culture plates for 7 days. The colony number was counted 7 days after plating under a reversed microscope. Aside from the colony count, the cell number was also counted to determine the colony size. Three independent experiments were carried out.

### Flow cytometric analysis

For analysis of cellular apoptosis, the transfected cells were stained using an antibody conjugate specific for APC Annexin V (BD Biosciences) with DAPI in the binding buffer following the manufacturer’s directions. Cells were incubated in the dark for 15 min at room temperature. The stained cells were detected *via* flow cytometric analysis. For each measurement, at least 10,000 cells were counted for cell cycle analysis, and BrdU incorporation and DAPI staining were performed according to the manufacturer’s guidelines (BD Biosciences, cat. 51–2090KZ). Briefly, cells were treated with 10 μM BrdU for 2 h. The cells were harvested and fixed in the Cytofix/Cytoperm buffer for 30 min on ice then washed with 1xBD Perm/Wash Buffer and centrifuged for 5 min at 300*g*. Cells were permeabilized in the Cytoperm Buffer Plus Buffer for 10 min on ice and after the wash step re-fixed with the Cytofix/Cytoperm buffer for 5 min on ice. After the wash step, the cells were treated with DNase (30 μg of DNase/10^6^ cells) for 1 h at 37 °C. Then, after washing, the cells were incubated with APC-conjugated anti-BrdU antibody (cat. 17–5071–42, eBioscience) for 20 min at room temperature, washed, and stained with DAPI to determine total DNA content.

For erythroid cell differentiation analysis, cells were stained with Glycophorin A (CD235a) and CD71 (transferrin receptor) antibodies. Cells were washed once in PBS supplemented with 2% FBS (PBS-FBS) and then resuspended in a 1:200 dilution of antibodies in PBS-FBS. The cells were incubated for 20 min at 4 °C in the dark, then washed twice with PBS-FBS. Data were acquired on BD CytoFLEX flow cytometer (Becton Dickinson) and analyzed using FlowJo 10 software (Tree Star Inc, Ashland, OR). These experiments were performed according to published procedures (49, 50).

### Quantitative reverse transcription real-time PCR (qRT-PCR)

Total RNA was extracted from cells using TRIzol Reagent (Life Technologies) and reverse transcribed using iScript Reverse Transcription Supermix (BioRad, 1708841) according to the manufacturer’s protocol. qRT-PCR was performed on a *QuantStudio 3* Real-Time PCR *System* (Applied Biosystems) using SsoAdvanced Universal SYBR Green Supermix (BioRad, 1725275). Reactions were performed in biological and technical triplicates using 12.5 ng of cDNA. The cycling conditions used were initial denaturation at 95 °C for 30 s, and 40 cycles of 95 °C for 15 s and 60 °C for 30 s. Primers were designed using Primer five and PrimerBank (http://pga.mgh.harvard.edu/primerbank/index.html) and are listed in Table S1. Transcript levels were normalized to glyceraldehyde-3- phosphate dehydrogenase (GAPDH) and fold changes were determined using the comparative Ct method. Student’s *t* test was performed for statistical significance.

### Western blot analysis

Total cellular proteins were extracted using RIPA Buffer (Auragene). Samples were boiled with 5X sample buffer (200 mM Tris (pH 6.8), 3.575 M β-mercaptoethanol, 10% SDS, 0.05% bromophenol blue, and 50% glycerol) for 12 min. Equal amounts of protein samples were resolved by 12% SDS-PAGE and then transferred onto PVDF membranes. The membranes were blocked with 5% non-fat milk at room temperature for 2 h, followed by incubation with primary antibodies at 4 °C overnight. Subsequently, membranes were incubated with an HRP-conjugated secondary antibody (Anti-rabbit or mouse IgG, HRP-linked antibody; 1: 5000) at room temperature for 2 h. To visualize the protein blots, an enhanced chemiluminescence (ECL) kit (Amersham Pharmacia) was used according to the manufacturer’s protocol. The following primary antibodies were used: Rabbit polyclonal anti-CTCF (D31H2, Cell Signaling Technology Inc., Beverly, MA, USA), TAF15 (25521-1-AP, Proteintech Group, Inc, USA), TCEB3 (Elongin A) (A300–942A, Bethyl Laboratories, Inc), TFII- I (sc-9943, Santa Cruz Biotechnologies), αE2F6 (sc-390022, Santa Cruz Biotechnologies), GRP78 (ab21685, Abcam) and β-actin (15G5A11/E2, Thermo Fisher Scientific Inc).

### Chromatin immunoprecipitation (ChIP) followed by qPCR

The ChIP assays were done in triplicate essentially as described by Stees *et al.* (51). 5 × 10^6^ cells were crosslinked and subjected to ChIP using IgG control or TFII-I specific antibodies. Incubation of cells with thapsigargin or H_2_O_2_ was done as described above for cell culture and stress induction. The purified DNA was subjected to qPCR using primers specific for the ATF4, CHOP, and necdin promoter region (see Table S1). Normal rabbit IgG (Sino Biological, CR1), and rabbit monoclonal anti-TFII-I (ChIP formulated, Cell Signaling Technology, 87604T) antibodies were used for the immunoprecipitation.

### Statistical analysis

All data were presented with standard deviation (SD+/−). Statistical significance was determined using the two-tailed Students *t* test. *p*-values with less than 0.05 were considered statistically significant. Statistical analysis was performed using GraphPad Prism 10 software.

## Data availability

All data are contained within the manuscript.

## Supporting information

This article contains supporting Information.

## Conflict of interest

The authors declare that they have no conflict of interest with the contents of this article.
