# Supplementary material for: TFII-I/GTF2I regulates globin gene expression and stress response in erythroid cells
Source: J Biol Chem. 2025 Jan 24;301(3):108227. doi: 10.1016/j.jbc.2025.108227 (PMC11879681; doi:10.1016/j.jbc.2025.108227)
Supplement: Supporting information [file mmc1.docx]

| **Gene** | **Forward primer ( 5' - 3' )** | **Reverse primer ( 5' - 3' )** |
| --- | --- | --- |
| GAPDH | GAAGGTGAAGGTCGGAGTCA | TTGAGGTCAATGAAGGGGTC |
| TFII-I | CAAGGAAGCCACCATTCTTC | TAGCTCATTGGCCTTTGGTC |
| CDC27 | ACTAAACCACTATGCTTACCGAGATG | CACTCCACCAGATAAGATTTGTTCC |
| CYCLIND1 | CCGTCCATGCGGAAGATC | GAAGACCTCCTCCTCGCACT |
| CDKNC1 | CAGCTGCACTCGGGGATTTC | GATCTCTTGCGCTTGGCGAAG |
| GADD45A | CTGGAGGAAGTGCTCAGCAAAG | AGAGCCACATCTCTGTCGTCGT |
| ATF4 | TCCGAATGGCTGGCTGTGG | GAAGACCTCCTCCTCGCACT |
| ATFJ1 | GGATTTTCAGCACCTTGCCC | TTGACAAAGGGCGTCAGGTT |
| ATFJ2 | CTCGGGGTGTCCATCACAAA | GGCACTCCGTCTTCTCCTTC |
| ATFJ3 | AGTGCCTGCAGAAAGAGTCG | AATACACGTGGGCCGATGAA |
| DNMT1 | AAGCAAGAAGTGAAGCCCGT | CTTAGCCTCTCCATCGGACT |
| HDAC1 | AGTGCGGTGGTCTTACAGTG | CCTCCCAGCATCAGCATAGG |
| GATA1 | AAACGGGCAGGTACTCAGTG | CGGTTCACCTGGTGTAGCTT |
| ATF3 | CTGCAGACTGAGAGCCCATC | CTTGGTTCAGGCTCAGAGGG |
| CHOP | CATCACCACACCTGAAAGCA | TCAGCTGCCATCTCTGCA |
| RAD21 | GGATAAGAAGCTAACCAAAGCCC | CTCCCAGTAAGAGATGTCCTGAT |
| TAL1 | AGGGCCTGGTTGAAGAAGAT | AAGTAAGGGCGACTGGGTTT |
| TAF15 | GATTCTGGAAGTTACGGTCAGTC | AGCTTTGTGATGCTTGTCCATAG |
| ELONGIN A | AACCCGGACCCTAAGAAGCTA | TCTCCGCAAGAATGTCTACTGTA |
| ALFA-GLOBIN | GAGGCCCTGGAGAGGATGTTCC | ACAGCGCGTTGGGCATGTCGTC |
| BETA-GLOBIN | TACATTTGCTTCTGACACAAC | ACAGATCCCCAAAGGAC |
| DELTA GLOBIN | GACTGCTGTCAATGCCCTGT | AAAGGCACCTAGCACCTTCTT |
| GAMMA GLOBIN | CTTCAAGCTCCTGGGAAATGT | GCAGAATAAAGCCTACCTTGAAAG |
| EPSILON GLOBIN | GCCTGTGGAGCAAGATGAAT | GCGGGCTTGAGGTTGT |
| ZETA GLOBIN | CCCGCAGACCAAGACCTAC | ACGACCGATAGGAACTTGTCC |
| ATF4 (ChIP) | CCGCCCACAGATGTAGTT | TGCAAAGGCCAATGCTG |
| CHOP (ChIP) | GGGCCAAGAAATATGGGAGT | CCTCTTTGCCGCTTGTCT |
| Necdin (ChIP) | GTGTTATGTGCGTGCAAACC | CTCTTCCCGGGTTTCTTCTC |

**Supplementary Table 1:** Oligonucleotide DNA primers used in quantitative PCR reactions.
